# Effective Caspase Inhibition Blocks Neutrophil Apoptosis and Reveals Mcl-1 as Both a Regulator and a Target of Neutrophil Caspase Activation

**DOI:** 10.1371/journal.pone.0015768

**Published:** 2011-01-06

**Authors:** David J. Wardle, Joseph Burgon, Ian Sabroe, Colin D. Bingle, Moira K. B. Whyte, Stephen A. Renshaw

**Affiliations:** 1 Medical Research Council Centre for Developmental and Biomedical Genetics, University of Sheffield, Sheffield, United Kingdom; 2 Department of Infection and Immunity, University of Sheffield, Sheffield, United Kingdom; University of Birmingham, United Kingdom

## Abstract

Human tissue inflammation is terminated, at least in part, by the death of inflammatory neutrophils by apoptosis. The regulation of this process is therefore key to understanding and manipulating inflammation resolution. Previous data have suggested that the short-lived pro-survival Bcl-2 family protein, Mcl-1, is instrumental in determining neutrophil lifespan. However, Mcl-1 can be cleaved following caspase activity, and the possibility therefore remains that the observed fall in Mcl-1 levels is due to caspase activity downstream of caspase activation, rather than being a key event initiating apoptosis in human neutrophils.

We demonstrate that apoptosis in highly purified neutrophils can be almost completely abrogated by caspase inhibition with the highly effective di-peptide caspase inhibitor, Q-VD.OPh, confirming the caspase dependence of neutrophil apoptosis. Effective caspase inhibition does not prevent the observed fall in Mcl-1 levels early in ultrapure neutrophil culture, suggesting that this fall in Mcl-1 levels is not a consequence of neutrophil apoptosis. However, at later timepoints, declines in Mcl-1 can be reversed with effective caspase inhibition, suggesting that Mcl-1 is both an upstream regulator and a downstream target of caspase activity in human neutrophils.

## Introduction

We are protected against infectious disease by a range of mechanisms, including innate immune cells such as neutrophils and macrophages. Neutrophils have the shortest lifespan of any healthy cell, and this brief lifespan limits pro-inflammatory functions of the neutrophil [1]. At sites of infection or potential infection (tissue injury), neutrophils are thought to have an extended functional lifespan [2], allowing the body to more evenly match neutrophil numbers with the numbers of rapidly dividing bacteria. Survival signals received by the neutrophils, such as cytokines (e.g. GM-CSF [3]), bacterial products [4] and hypoxia [5] act to profoundly delay neutrophil lifespan in *in vitro* cultures, which are thought to mimic the *in vivo* behaviour of neutrophils. Importantly, the downstream molecular signals by which these survival signals impinge on the pathways governing the normally short lifespan of the neutrophil are incompletely known.

It is generally accepted that multidomain pro-survival Bcl-2 family proteins are important in regulating neutrophil longevity [6]. Mcl-1 [7] and, to a lesser extent, A1 [8] have been shown to be important for maintaining neutrophil survival, and have additionally been implicated in signalling extended neutrophil lifespan in response to a variety of stimuli including cytokines [9], elevated cAMP [10] and hypoxia [11], [12]. A key role for Mcl-1 is further supported by the decreased survival seen in myeloid cells treated with antisense oligonucleotides against Mcl-1 [12], [13]. Increased Mcl-1 levels have also been reported in inflammatory settings *in vivo*: in freshly isolated neutrophils from Crohns disease patients [14]; and in animal models of endotoxaemia [15]. Importantly, therapeutic strategies using CDK inihibitors or Lipoxins to target neutrophils appear to act via modulation of Mcl-1 [16]–[18].

Mcl-1 is regulated in complex ways, with multiple isoforms generated by alternative splicing present in human neutrophils [19]. In addition, Mcl-1 is known to be proteolytically cleaved downstream of caspase activity, leading to multiple protein products on Western blotting [20]. Caspase activity has been demonstrated in human neutrophils, but the role this plays in determining the timing of neutrophil death is controversial, with apparently conflicting data in the literature [21]–[24].

Some observers have surmised that falling Mcl-1 levels initiate caspase activation [6] by removal of inhibition of pro-apoptotic Bcl-2 family proteins, such as Bim. In this model, BH-3 only proteins act to cause cytochrome c release from the mitochondria, and hence Apaf-1 dependent caspase activation. Published data show an inverse relationship between Mcl-1 levels and degree of apoptosis in all neutrophil populations studied. This could either indicate that falling Mcl-1 levels trigger apoptosis, or Mcl-1 is degraded by apoptotic proteases – a well described phenomenon [20], [25]. We hypothesised that new and potent caspase inhibitors might allow us to dissociate falls in Mcl-1 levels from rates of neutrophil apoptosis. We show that the irreversible dipeptide caspase inhibitor Q-VD.OPh (quinolyl-valyl-O-methylaspartyl-[-2,6-difluorophenoxy]-methylketone) profoundly inhibits neutrophil apoptosis, and that Mcl-1 levels drop in advance of apoptosis, even in the presence of caspase inhibition. At later timepoints, however, Mcl-1 levels are inversely proportional to the fraction of apoptotic neutrophils in the population. These data suggest Mcl-1 is both a regulator and a downstream target of caspase activation in the neutrophil.

## Methods

### Ethics Statement

Peripheral venous blood was taken from healthy volunteers in accordance with the specific approval of the South Sheffield Research Ethics Committee (reference number: STH13927). Fully informed written consent was obtained, and all clinical investigation was conducted according to the principles expressed in the Declaration of Helsinki.

### Reagents

All reagents including zVAD.fmk were from Sigma-Aldrich (Poole, UK) unless otherwise stated. Q-VD.OPh was from R&D systems (Abingdon, UK). Recombinant human GMCSF was from Peprotech (London, UK).

### Purification of peripheral blood neutrophils

Percoll blood preparations were performed as previously described [26]. Briefly, Dextran sedimented neutrophils were separated by centrifugation over a plasma-Percoll column, washed and resuspended in RPMI, with 10% Foetal Calf Serum (Invitrogen) and 100 U/ml of Penicillin and 100 µg/ml Streptomycin (Life Technologies) at 5×10^6^ neutrophils per ml. The OptiPrep™ (Axis-Shield, Upton, Huntingdon, UK) method of neutrophil isolation is a modification of the Percoll method using an OptiPrep™ gradient rather than Percoll. Cells isolated with the OptiPrep™ method were further purified by negative magnetic selection using a custom cocktail (Stemcell Technologies, Vancouver, Canada) [4]. Cytospin preparations fixed with 100% methanol and stained with Quick-Diff (Gentaur) were counted using a TE-2000U microscope (Nikon, Tokyo, Japan) at ×900 magnification. Neutrophil, eosinophil and PBMC numbers were counted, based on their characteristic appearance. A total of 500 cells were counted per sample, and the proportion of neutrophils to eosinophils and PBMC was noted. For assessment of apoptosis 300 neutrophils were counted by a blinded, trained observer.

### Detection of neutrophil Mcl-1 by western blot analysis

Neutrophils were resuspended in ice-cold phosphate buffered saline (PBS) solution and centrifuged at 300 g for 2 minutes. Neutrophils were then resuspended in 30 µl of ice cold buffer (50 mM Tris, 50 mM NaF, 50 mM β-glycerophosphate, 10 mM Sodium Orthovanadate) with 1∶100 protease inhibitor cocktail set IV and 1 mM PMSF. This was immediately followed by the addition of 30 µl ice cold phosphatase lysis buffer (buffer above plus 1 in 50 Triton-X100), also with 1∶100 protease inhibitor cocktail set IV and 1 mM PMSF. 60 µl of 2xSDS lysis buffer (1 M DTT, 20% SDS, Glycerol, 0.5 M pH 6.8 Tris-HCl, 0.2% Bromophenol Blue) with 1∶50 EDTA free protease inhibitor cocktail was then added and the sample was heated at 95°C for 10 minutes. The samples were frozen at −80°C until required for analysis. Western blotting was performed according to standard protocols [27], using Protean 2 gel assembly (Bio-Rad, Berkeley, CA). Protein samples from 1 to 1.5×10^6^ neutrophils were run alongside 10 µl of full-range rainbow molecular weight ladder (GE Healthcare Life Sciences, Uppsala, Sweden), and transferred to Hybond™-P PVDF membrane (GE Healthcare Life Sciences) using a Trans-Blot® semi dry blotter (Bio-Rad). Antibody incubations were overnight at 4°C (primary) and 60 minutes at room temperature (secondary).

### Quantification of western blot band density

Films were scanned using an Epson flatbed scanner in TIFF format and densitometry was performed using Image J [28], according to online instructions [29]. Band density was given as a percentage of the overall density of all bands, making the measurement relative. To eliminate loading discrepancies, this measurement was then given as a percentage of the density of the actin control band, to give the final relative protein expression.

## Results and Discussion

### Neutrophil apoptosis is profoundly inhibited by the caspase inhibitor Q-VD.OPh

**Figure 1:**
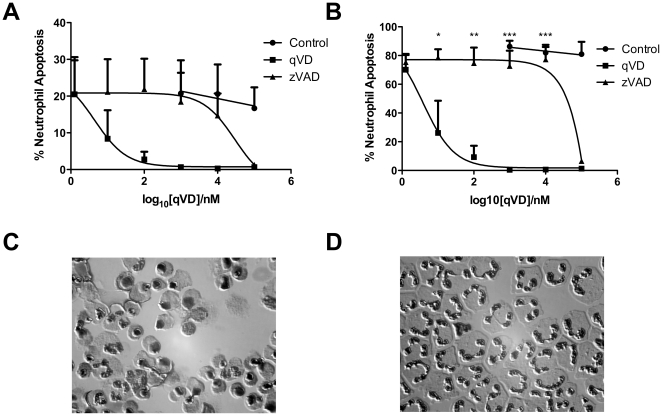
The caspase inhibitor Q-VD.OPh profoundly inhibits neutrophil apoptosis. **A,B**) Neutrophils were cultured for A) 8 hours, and B) 20 hours with and without the pan-caspase inhibitors zVAD.fmk and Q-VD.OPh with a DMSO vehicle control (n = 4). Q-VD.OPh dramatically decreased neutrophil apoptosis from 10 nM, whereas no effects of zVAD were seen below 100 µM. *P<0.05, **P<0.01, ***p<0.001 for comparison of Q-VD vs zVAD, one way ANOVA with Bonferroni's post-test correction, comparing all values. **C**) Cytospin preparation of neutrophils in control conditions after 20 hours. Apoptotic cells are clearly visible by their condensed nuclei. **D**) Cytospin preparation of neutrophils cultured with Q-VD.OPh after 20 hours. No apoptotic cells can be seen and neutrophils exhibit the classic polymorphic nuclear morphology of a healthy neutrophil. Images were taken using a 60× Plan Apo Oil immersion NA1.40, Nikon, with 1.5× magnification on a Nikon TE2000U.

The caspase dependency of neutrophil apoptosis remains controversial. Several publications have indicated that caspase inhibitors are ineffective in blocking neutrophil apoptosis, and have concluded from this that caspase independent apoptosis pathways are implicated in limiting neutrophil lifespan [22], [23], [30]. A recent review reflected this uncertainty [24]. However, our own work has supported a role for caspase inhibitors in delaying neutrophil apoptosis [21]. In order to test whether the differences in the literature are due to the different caspase inhibitors and concentrations used, we performed a detailed dose response analysis of the most commonly used caspase inhibitor in neutrophil studies, zVAD.fmk against a new dipeptide inhibitor which we had found to be profoundly effective in delaying apoptosis in neutrophils, Q-VD.OPh. This pan-caspase inhibitor, like zVAD.fmk, binds irreversibly to the active site of activated caspases. It is non-toxic to cells, highly cell permeable and has been found to be more effective at reducing apoptosis than zVAD.fmk [31]. zVAD.fmk does not inhibit neutrophil apoptosis at concentrations below 100 µM (Figure 1), suggesting the ineffectiveness of zVAD.fmk in previously published studies may be a feature of the higher concentrations required in neutrophils compared to other cell types. In contrast, Q-VD.OPh is highly effective at blocking neutrophil apoptosis at concentrations as low as 100 nM, and begins to have an effect at 10 nM. Therefore, these data present Q-VD.OPh as a highly effective neutrophil caspase inhibitor and confirm the requirement for caspase activity in neutrophil apoptosis.

Q-VD.OPh differs from other caspase inhibitors in three distinct ways that might contribute to its increased potency and efficacy in inhibiting neutrophil apoptosis. As a dipeptide inhibitor it is smaller and may pass more easily into cells. The process of cell penetration may be further enhanced by the addition of the N-terminal quinolyl group. Finally, the carboxy terminal O-Phenoxy group replaces the fluoromethylketone group, and has been reported as reducing toxicity [31].

### At early timepoints, Mcl-1 levels fall even in the presence of highly effective caspase inhibition

**Figure 2:**
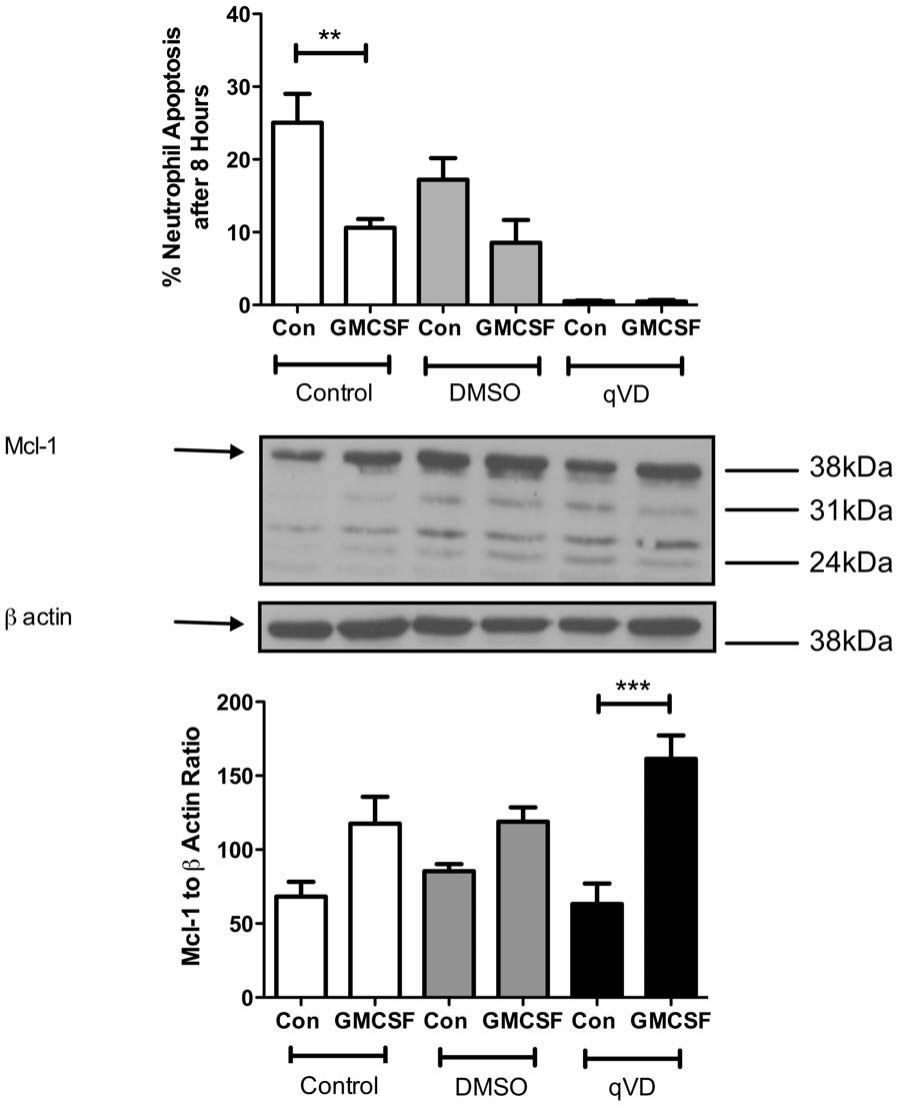
At early timepoints, Mcl-1 levels fall even in the presence of highly effective caspase inhibition. Neutrophils were cultured ± caspase inhibitor, Q-VD.OPh (QVD), ± DMSO (vehicle control) for 8 hours. Neutrophil apoptosis rates were determined by cytospin analysis (upper panel), and relative Mcl-1 levels determined using western blotting for 3 independent experiments (middle panel). Q-VD.OPh significantly reduced rates of apoptosis. In the presence of Q-VD.OPh, amounts of Mcl-1 were significantly lower in control neutrophil lysates compared to lysates from GMCSF treated cells (*p<0.001 for control vs GMCSF, one way ANOVA with Bonferroni's post-test correction, n = 3.)

One of the key unresolved questions in neutrophil biology is the relationship between neutrophil apoptosis and falling Mcl-1 levels. Mcl-1 levels fall as apoptosis progresses, and survival signals that prolong neutrophil lifespan increase Mcl-1 levels [9]. However, Mcl-1 is also a well-recognised caspase substrate in various cell types [20], [25], [32], [33], including neutrophils in certain circumstances [34]. Therefore it is important to identify whether Mcl-1 degradation is the signal for apoptosis and caspase activity, or whether it is degraded downstream of caspases in neutrophils. Previous studies have used lower doses of caspase inhibitors in the presence of cyclohexamide [35]. We therefore tested the ability of highly effective caspase inhibition to maintain Mcl-1 levels in neutrophils in the absence of protein synthesis inhibitors. Mcl-1 levels at 8 hours were not altered by highly effective caspase inhibition with Q-VD.OPh (Figure 2), and GM-CSF was able to significantly increase Mcl-1 levels, back to levels comparable to those in freshly isolated neutrophils (Figure S1). These data confirm previous suggestions that, at early timepoints, the observed reduction in Mcl-1 levels is an upstream event in initiating caspase activity and apoptosis.

### At later timepoints, Mcl-1 degradation is downstream of caspase activation in neutrophils

**Figure 3:**
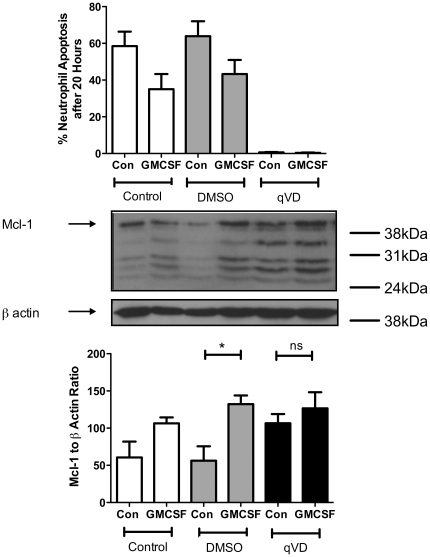
At late timepoints, Mcl-1 levels are maintained by highly effective caspase inhibition. Neutrophils were cultured ± caspase inhibitor, Q-VD.OPh (QVD), ± DMSO (vehicle control) for 20 hours. Neutrophil apoptosis rates were determined by cytospin analysis (upper panel), and relative Mcl-1 levels determined using western blotting for 3 independent experiments (middle panel) as previously shown. In the presence of Q-VD.OPh, there was no significant difference in levels of Mcl-1 in control neutrophil lysates compared to lysates from GMCSF treated cells (*p<0.001 for control vs GMCSF, one way ANOVA with Bonferroni's post-test correction, n = 3.)

Rates of apoptosis are low at early timepoints, and effective caspase inhibition has profound and long-lasting effects, still completely blocking neutrophil apoptosis at 20 hours. We therefore investigated the effect of complete blockade of caspase dependent neutrophil apoptosis with Q-VD.OPh on Mcl-1 levels at later timepoints. Mcl-1 levels were increased in neutrophils treated with Q-VD.OPh alone, to the same level as GMCSF treated neutrophils and freshly isolated neutrophils (Figure S1), suggesting that by 20 hours the observed decrease in Mcl-1 levels are in part a consequence of caspase activation, and caspase-dependent Mcl-1 degradation (Figure 3). In other words, at later timepoints the decrease in Mcl-1 levels appears to be a consequence of apoptosis, rather than a controlling factor. This would imply that GM-CSF is able modulate apoptosis via Mcl-1 independent mechanisms, as also suggested by the ability of GM-CSF to delay neutrophil apoptosis in mice with a myeloid specific Mcl-1 defect [7].

Since Mcl-1 is a known caspase substrate in some cells, it might be expected that the increase in Mcl-1 levels induced by caspase inhibition occurs via directly protecting Mcl-1 from caspase cleavage. However, unlike other groups [20], we did not detect Mcl-1 caspase-cleavage products in our control samples using the same anti-Mcl-1 antibody. The additional bands identified were larger than Mcl-1 caspase cleavage fragments and are thought to be Mcl-1 splice variants [19]. Expression of these bands, both in absolute terms and as a proportion of full-length Mcl-1, was not significantly altered, even by complete suppression of caspase-dependent cell death by Q-VD.OPh (Figures 2 and 3). Therefore, this suggests that Mcl-1 degradation at 20 hours might be mediated downstream of caspase activation, possibly by calpains [36], rather than by caspases directly.

### GM-CSF acts directly on neutrophils to increase Mcl-1 protein levels

**Figure 4:**
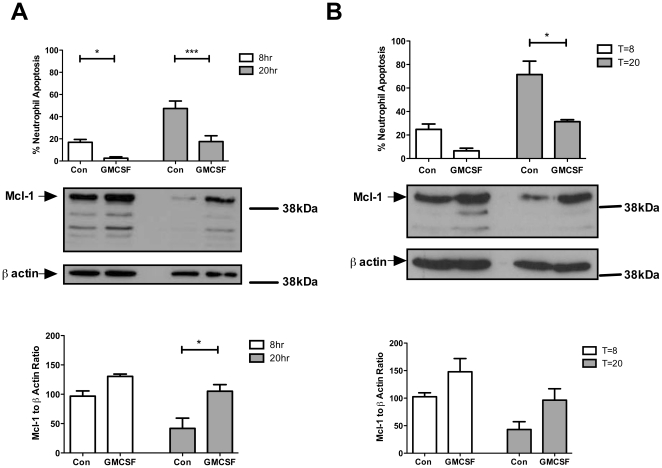
GM-CSF increased Mcl-1 levels in standard purity and highly purified neutrophils between 8 and 20 hours. **A**. Rates of apoptosis (top) and Mcl-1 expression ± GM-CSF as determined by western blot analysis in neutrophils isolated using the Percoll method. n = 3 independent experiments from different donors. *P<0.05, *** p<0.001, one-way ANOVA, with Bonferroni's post-test correction for multiple comparisons. **B**. Rates of apoptosis (top) and Mcl-1 expression ± GM-CSF as determined by western blot analysis in neutrophils isolated using the OptiPrep (+NMS) method. n = 3 independent experiments from different donors. *P<0.05 one-way ANOVA, with Bonferroni's post-test correction for multiple comparisons.

Isolation of granulocytes from other haematological cells is usually based on density, with separation using a discontinuous plasma/Percoll™ density gradient being a widely used method [26], [37]. This method usually results in high levels of granulocyte purity, with 1–2% contaminating Peripheral Blood Mononuclear Cells (PBMC). Density-based separation methods cannot distinguish between eosinophils and neutrophils, therefore overall neutrophil purity is dependent on donor eosinophil numbers. Contaminating mononuclear cells are able to profoundly influence the behaviour of neutrophils in culture [38], and may contribute disproportionate levels of certain proteins to western blotting analysis. It has previously been shown that, unlike LPS, GM-CSF delay of neutrophil apoptosis is not dependent on contaminating monocytes [39]. However, the response of Mcl-1 to survival signals such as GM-CSF might relate to either induction of Mcl-1 in neutrophils by unspecified monocyte factors, or to direct monocyte contamination of protein lysates. Levels of Mcl-1 and modulation by GM-CSF were therefore assessed in standard purity neutrophil preparations and in ultrapure neutrophil preparations (with negative magnetic selection), resulting in small and no PBMC contamination respectively (1.4%±0.48 n = 6 vs 0.0±0.0 n = 4). These cells were cultured with and without GM-CSF for 8 and 20 hours, after which apoptosis rates were determined by cytospin analysis, and compared to Mcl-1 levels assessed by comparative densitometry of three independent western blot analyses. In standard purity neutrophil cultures, Mcl-1 levels declined between 8 and 20 hours in control neutrophils, as has been previously shown [9]. Mcl-1 was upregulated in GM-CSF stimulated neutrophils compared to control cells at both 8 and 20 hours, showing statistical significance by 20 hours in Percoll purified samples (Figure 4A). Reduction in Mcl-1 levels over time and inhibition of this reduction by GMCSF are seen in the ultrapure preparations at a comparable level (Figure 4B), suggesting that the effects of GMCSF on Mcl-1 levels are not due to the influence of contaminating monocytes.

### Conclusion

Regulation of Mcl-1 levels within human neutrophils has key effects on neutrophil survival *in vivo* [7] and *in vitro* [9]. Mcl-1 levels initially fall during neutrophil culture *in vitro* independently of neutrophil apoptosis, and these data support the assertion that this fall in Mcl-1 levels may be the initiating event of neutrophil apoptosis. However, by 20 hours of culture, Mcl-1 levels reflect degree of apoptosis, suggesting that at these timepoints Mcl-1 levels are predominantly regulated by degradation downstream of caspases.

## Supporting Information

Figure S1 **Mcl-1 levels fall between 0 and 8 hours in culture and GM-CSF prevents this reduction.** **A**. Neutrophils were either lysed at time 0 or cultured ± GM-CSF for 8 hours and relative Mcl-1 levels determined using western blotting. Mcl-1 levels fall significantly between time 0 and time 8 lysates (*p<0.001 for control vs 8 hours - GMCSF, one way ANOVA with Bonferroni's post-test correction, n = 2.). GM-CSF treatment maintains Mcl-1 levels.
(TIF)
