# Supplementary figures and images for: Effective Caspase Inhibition Blocks Neutrophil Apoptosis and Reveals Mcl-1 as Both a Regulator and a Target of Neutrophil Caspase Activation
Source: PLoS One. 2011 Jan 6;6(1):e15768. doi: 10.1371/journal.pone.0015768 (PMC3017075; doi:10.1371/journal.pone.0015768)

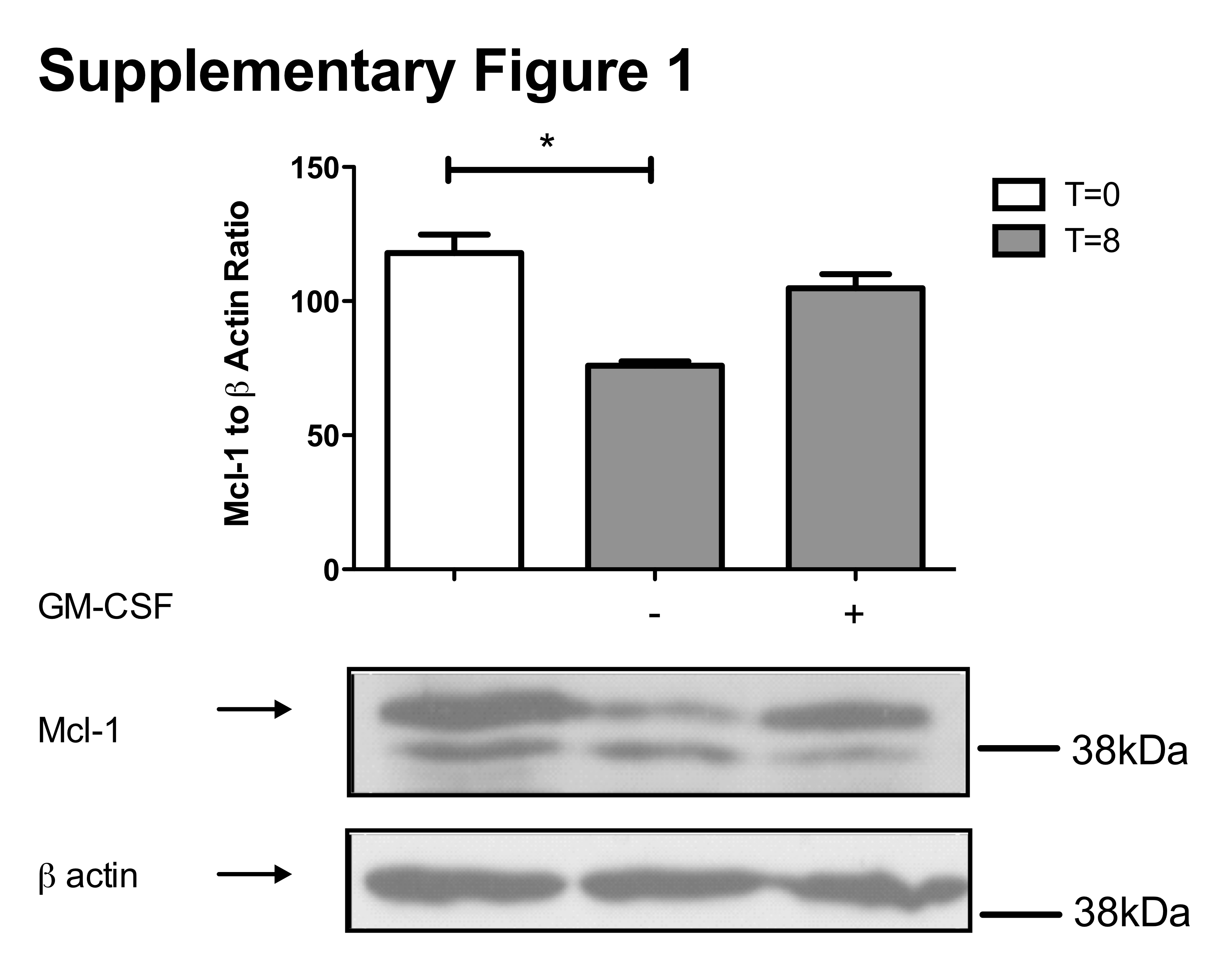

Supplement: Figure S1 — Mcl-1 levels fall between 0 and 8 hours in culture and GM-CSF prevents this reduction. A. Neutrophils were either lysed at time 0 or cultured ± GM-CSF for 8 hours and relative Mcl-1 levels determined using western blotting. Mcl-1 levels fall significantly between time 0 and time 8 lysates (*p<0.001 for control vs 8 hours - GMCSF, one way ANOVA with Bonferroni's post-test correction, n = 2.). GM-CSF treatment maintains Mcl-1 levels. (TIF) [file pone.0015768.s001.tif]
